# Are Happy Faces Attractive? The Roles of Early vs. Late Processing

**DOI:** 10.3389/fpsyg.2015.01812

**Published:** 2015-11-30

**Authors:** Delin Sun, Chetwyn C. H. Chan, Jintu Fan, Yi Wu, Tatia M. C. Lee

**Affiliations:** ^1^Laboratory of Neuropsychology, The University of Hong KongHong Kong, China; ^2^Applied Cognitive Neuroscience Laboratory, The Hong Kong Polytechnic UniversityHong Kong, China; ^3^Department of Fiber Science and Apparel Design, Connell UniversityNew York, NY, USA; ^4^Department of Rehabilitation Medicine, Huashan Hospital, Fudan UniversityShanghai, China; ^5^Institute of Clinical Neuropsychology, The University of Hong KongHong Kong, China

**Keywords:** face, attractiveness, expression, ERP, P2, LPP

## Abstract

Facial attractiveness is closely related to romantic love. To understand if the neural underpinnings of perceived facial attractiveness and facial expression are similar constructs, we recorded neural signals using an event-related potential (ERP) methodology for 20 participants who were viewing faces with varied attractiveness and expressions. We found that attractiveness and expression were reflected by two early components, P2-lateral (P2l) and P2-medial (P2m), respectively; their interaction effect was reflected by LPP, a late component. The findings suggested that facial attractiveness and expression are first processed in parallel for discrimination between stimuli. After the initial processing, more attentional resources are allocated to the faces with the most positive or most negative valence in both the attractiveness and expression dimensions. The findings contribute to the theoretical model of face perception.

## Introduction

Romantic love is closely associated with mate choice (Fisher et al., 2005), in which facial attractiveness plays a critical role (Little et al., 2011; Little, 2014). The effect of facial attractiveness has been found to be modulated by facial expression. For example, a smile was evaluated as more attractive than a neutral expression (Otta et al., 1996), and the preference for attractive faces was enhanced by happy expressions (Main et al., 2010). However, the classical theoretical models of face perception (Bruce and Young, 1986; Haxby et al., 2000) do not clarify whether the perceptions of attractiveness and expression are processed in similar ways. This question has become an important research objective given the increased interest in investigating the relationship between facial attractiveness and romantic love.

It is speculated that perception of attractiveness and expression share similar processing, given that both attractiveness and expression are derived from facial characteristics (e.g., size, position and movement of eyes, nose and mouth) and that both are capable of eliciting affective experiences in the observers. Besides, previous functional magnetic resonance imaging (fMRI) studies have shown that a number of occipital, limbic, temporal, parietal, and prefrontal brain regions that responded to the manipulation of attractiveness (O'Doherty et al., 2003; Ishai, 2007; Winston et al., 2007; Chatterjee et al., 2009) responded to the manipulation of expression (Vuilleumier and Pourtois, 2007; Fusar-Poli et al., 2009). Moreover, O'Doherty et al. (2003) found that the increased activation elicited by faces of high attractiveness in the orbitofrontal cortex (OFC), a reward-related brain region, was enhanced by a smile, suggesting that some common neural processing (e.g., reward) is shared by perceptions of attractiveness and expression. The aforementioned speculation is still controversial. Previous studies have proven that the processing of face perception is hierarchical; for reviews, please see Eimer and Holmes (2007) and Olofsson et al. (2008). It is possible that attractiveness and expression are processed separately and then are processed as a whole, even if similar brain areas are involved for processing the two types of facial information. However, the fMRI's poor temporal resolution (i.e., 2–3 s) makes it difficult to delineate the time course of face perception's quick neural processing.

The event-related potential (ERP) methodology has a high temporal resolution (i.e., a few milliseconds) and has been widely used to examine the neural correlates of face perception in the temporal domain. Previous ERP studies have revealed a few correlates of perceiving either attractiveness or expression. Firstly, the P2 component is a positive-going deflection at the frontal or parietal sites, peaking at around 200 ms; it is supposed to reflect the comparison between sensory input and stored memory (Luck and Hillyard, 1994) and initial “attention capture” of (physically) distinctive faces (van Hooff et al., 2011). The role of P2 in perceiving facial attractiveness has been reported. An early study by Halit et al. (2000) showed that stretching the distance between pupil, nose and lip made a face less attractive, compared to its original image, and this alteration elicited larger P2 for attractive (intact) faces than unattractive (stretched) faces. van Hooff et al. (2011) found that both attractive and unattractive faces elicited larger P2 peak amplitudes at Pz channel within 120–220 ms than faces with medium ratings of attractiveness. Zhang and Deng (2012) found larger P2 within 150–230 ms for attractive than unattractive faces at CPz and Pz channels. On the other hand, previous studies have shown that P2 responds to the emotional content of stimuli, although the polarity of contrast varied across studies. For example, Spreckelmeyer et al. (2006) showed that P2 was more pronounced for happy (vs. neutral and sad) pictures and for pictures paired with happy (vs. neutral and sad) voices. In contrast, Ofsson and Polich (2007) found that unpleasant pictures yielded larger P2 than did neutral and pleasant pictures.

Secondly, previous studies have also reported that a late component, late posterior positivity (LPP), responds to face stimuli. LPP typically appears in posterior sites after at least 350 ms and lasts for several hundred milliseconds, and it is supposed to reflect the facilitated attention allocation to motivationally relevant, emotional stimuli (Foti et al., 2009) and task-related evaluative processes (Johnston and Oliver-Rodriguez, 1997; Cuthbert et al., 2000; Werheid et al., 2007). Attractive faces were often found to elicit larger LPP than unattractive faces (Johnston and Oliver-Rodriguez, 1997; Werheid et al., 2007; van Hooff et al., 2011; Zhang and Deng, 2012), even when the subjects were instructed to fake their responses (Dong et al., 2010). However, a U-shaped relationship between the LPP amplitudes and the mean attractiveness ratings was also reported, reflected by larger LPP for attractive and unattractive faces than for faces of medium attractiveness (Schacht et al., 2008; Marzi and Viggiano, 2010). On the other hand, detected LPP was larger for fearful than for happy faces, and for happy than for neutral faces, in one study (Luo et al., 2010); larger LPP was also found for happy and neutral schematic expressions than for sad schematic faces in another study (Liu et al., 2013).

The mixed ERP findings listed above might be due to the varied task paradigms, visual stimuli and participants employed across studies. For example, some previous studies manipulated attractiveness while controlling expression, but other studies manipulated expression while controlling attractiveness. Here, we examined within the same group of participants the neural correlates of perceiving either attractive or unattractive faces with either happy or sad expressions. If attractiveness and expression are processed in the same way, we hypothesized that we would detect their interaction effect in P2 and LPP. Otherwise, if they are processed separately first and then integrated for processing, we hypothesized that we would detect the effects of attractiveness and/or expression in P2—and their interaction in LPP.

## Materials and methods

### Participants

Twenty university students (10 female and 10 male; mean age = 23.9 years, standard deviation [*SD*] = 7.5 years) were recruited from The Hong Kong Polytechnic University. All were right-handed (Oldfield, 1971), with normal or corrected-to-normal vision. None reported a history of physical, neurological, or mental disorders. All participants provided written informed consent. Ethics approval of this study was obtained from the local Institutional Review Board.

### Stimuli

**Figure 1:**
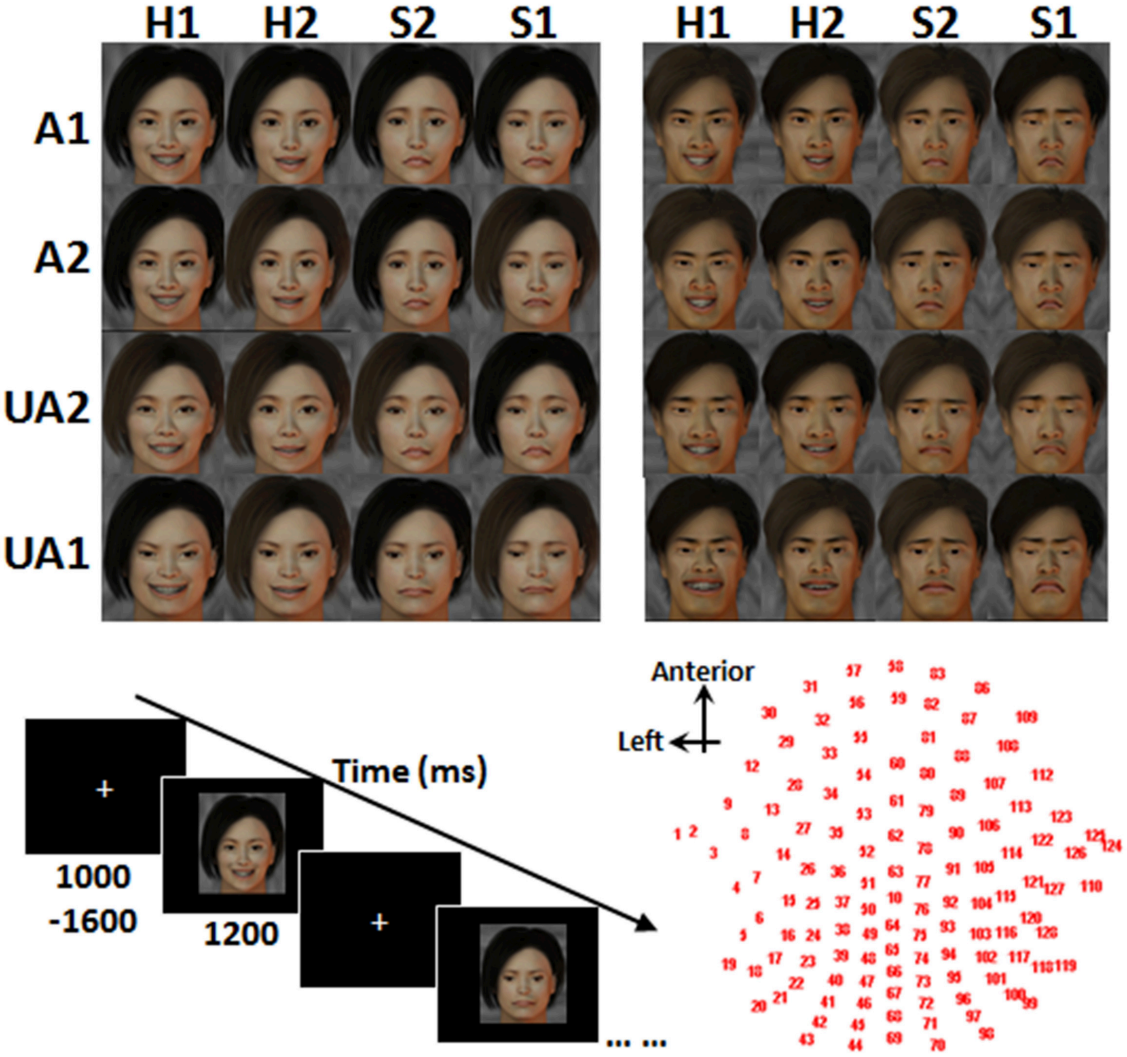
**Face stimuli, task paradigm and EEG channel locations**. A1, attractive; A2, less attractive; UA2, less unattractive; UA1, unattractive; H1, happy; H2, less happy; S2, less sad; S1, sad.

Photos of real human faces have often been utilized in previous studies on face perception. They are of high ecological validity, but their confounding factors (e.g., hair color/style, glasses, skin color/texture and sizes/positions of eyes, nose, mouth, and ears) are difficult to control for. Here, we employed fabricated facial stimuli, which were adapted from a few standard face templates, to control the variables of no interest. Facial stimuli were fabricated in two phases. The first phase was to generate face templates with varied levels of attractiveness, while the second phase was to integrate the different levels of facial expressions for forming the final stimuli. In the first phase, 32 Chinese faces (16 females and 16 males) with varied levels of attractiveness were generated with FaceGen software (FaceGen Modeler v3.4). All the faces were of front view with eyes gazing forward. The skin color, hair color, and illumination were also adjusted to the same level across stimuli. The validity of these 32 stimuli was verified by asking a different group of 20 participants to assign ratings reflecting the levels of attractiveness. Faces with the highest (attractive, or A1) and the lowest (unattractive, or UA1) mean ratings, and those with ratings in the 66th percentile (less attractive, or A2) and 33rd percentile (less unattractive, or UA2), were chosen. This resulted in 4 male and 4 female facial templates. In the second phase, happy (H1), less happy (H2), less sad (S2), and sad (S1) expressions were modeled to each of the facial templates according to the criteria described by Ekman (2003), which include raising (or lowering) the tails of both eyes and the edges of the mouth for a happier (or sadder) expression. This produced 32 facial stimuli (8 stimuli × 4 expressions, see Figure 1) for use in the main study.

### Task and procedure

The participant sat in front of a desktop computer, which was situated inside a sound-proof chamber. At the beginning of each trial, a white cross was presented at random intervals (1000–1600 ms), and a facial stimulus was presented on the computer screen for 1200 ms. Upon presentation of the stimulus, the participant was asked to attend to the attractiveness of the face in the Attraction condition and to the expression of the face in the Emotion condition. The participant was to judge the Level of Attractiveness in the Attraction condition or the Level of Expression in the Emotion condition. In most of the trials, the participants were asked to keep the judgments on each face to themselves. Participants were not required to indicate their judgments by making a physical response. This was to enable participants to pay attention to the perception and judgment of the faces and to avoid fatigue from the physical response. The participant, however, was required to make a physical response in each catch trial, which appeared once every 6 to 10 trials within a block. Instead of keeping the judgment to oneself, the participant was to undergo judgment of the face presented and respond using the right hand to press one of two buttons on a keyboard placed in front of the participant. The participant was instructed to regard both A1 and A2 faces as “attractive” and both UA2 and UA1 faces as “unattractive.” By the same token, H1 and H2 faces were regarded as “happy,” and S1 and S2 faces were regarded as “sad.” The right index finger was to press on the left button (indicating “attractive” or “happy”), and the right middle finger was to press on the right button (indicating “unattractive” or “sad”). The correspondence of the buttons to the responses was counterbalanced across the participants.

Stim2 software (Neuroscan Company) was employed to present the facial stimuli and to collect the responses. The 32 facial stimuli were presented twice in a randomized order in each block. There were a total of 16 blocks, eight for each of the Attraction and Emotion conditions. These gave a total of 1024 trials. Each block lasted for about 5 min, with a 30-s break between consecutive blocks. The order of the Attraction and Emotion conditions was interleaved for each participant and counterbalanced across the participants. Each participant was informed about the condition and reminded of the response set prior to beginning each block. Before commencing the experimental task, the participant completed 10 training trials in which the stimuli were different from those used in the formal task.

### Behavioral responses

For the behavioral data analyses, the faces were classified into 4 categories, i.e., the combinations of attractive (both A1 and A2)/unattractive (both UA1 and UA2) and happy (both H1 and H2)/sad (both S1 and S2) faces. Two 2 (attractiveness: attractive vs. unattractive) by 2 (expression: happy vs. sad) repeated measures ANOVA were conducted under the Attraction condition and the Emotion condition, respectively. *Post-hoc* analyses with Bonferroni correction were carried out if there is significant interaction effect.

### ERP data recording and preprocessing

Electroencephalogram (EEG) signals were recorded by a 128-channel fabric cap (Neuroscan company) embedded with Ag-AgCl electrodes in which impedances were kept below 5 kΩ. All channel recordings were referenced to a channel at the left mastoid. The EEG signals were amplified using a 0.01–200 Hz band-pass filter and continuously sampled at 1000 Hz. Vertical eye movement was recorded by two electrodes placed on the top and bottom parts of the left eye. Horizontal eye movement was monitored by two electrodes at the outer canthi of the two eyes.

The EEG data were preprocessed with Scan 4.3 software (Neuroscan Company). The signals were re-referenced to a computed average of channels over the whole scalp and filtered by 0.1–30 Hz band-pass using a zero phase-shift digital filter. Eye-blink artifacts were mathematically corrected (Gratton et al., 1983). Continuous signals were cut into −200 to 1000 ms epochs, with time 0 ms as the reference for the onset of the face stimulus. Baseline correction and artifact rejection were performed so that any signals exceeding ± 100 μV in any given epoch were discarded. The ERPs of each event for each participant were then averaged.

### ERP data statistical analysis

A two-pronged approach was employed for the ERP data analyses: (a) the whole scalp × time space analysis which provides conservative outputs but has the potential to identify unexpected findings, and (b) the region of interest (ROI) analysis which is more sensitive to task effects within sites and time windows selected a priori based on the literature. Here, both analyses were conducted by the statistical parametric mapping (SPM) method which does statistical comparisons voxel-by-voxel. A voxel here is a unit combining both spatial and temporal information (Litvak et al., 2011).

Statistical analyses were conducted using SPM12 software (Wellcome Department of Cognitive Neurology, London, UK). The ERPs were down-sampled to 200 Hz and were then converted into three-dimensional images through interpolating the ERP amplitude at each channel site per time point. The *x* and *y* dimensions of an image reflect “left-right” and “anterior-posterior,” respectively, on the horizontal projection of a scalp, and the z dimension represents the timeline. The images were smoothed by a full width at half-maximum (FWHM) Gaussian filter of 9 mm, 9 mm, and 20 ms (Sun et al., 2012, 2015). These images were entered into a Three-way analysis of variance (ANOVA) model in SPM12. The three factors were condition (two levels: Attraction and Emotion), Level of Attractiveness (four levels: A1, A2, UA2, and UA1) and Level of Expression (four levels: H1, H2, S2, and S1).

Voxel-by-voxel analyses within a large scalp × time space have to correct a large number of comparisons, and may decrease the statistical sensitivity to the task effects. We thus tried to reduce the number of voxels for comparisons. Firstly, an inclusive mask covering the whole scalp and the time range from 80 to 980 ms was employed to restrict the space of the analysis. This time interval has been commonly adopted in previous ERP studies on face perception. Secondly, some previous studies have reported very early ERP components responsive to attractiveness or expression. Here, to investigate the potential neural correlates before the peak of P2, a separate ANOVA was conducted for signals captured within the 100–200 ms time window.

The significant ANOVA results for clusters of voxels were height-thresholded at *p* < 0.001 (*F*-tests, two-tailed) and that survived peak- or cluster-level familywise error (FWE) correction (*p* < 0.05) within the space of analysis. Furthermore, the clusters showing significant main or interaction effects were employed as mask images to restrict the space of *post-hoc* analyses. In this study, the *post-hoc* analyses on the Level of Attractiveness/Level of Expression effect had six pair-wise effects (i.e., [4 – 1] × 2, since each had two contrast directions); while the number of *post-hoc* analyses on the Level of Attractiveness × Level of Expression effect was multiplied fourfold. The peak-level FWE *corrected p*-values after Bonferroni adjustments were 0.05/12 = 0.004 or 0.05/48 = 0.001, depending on the effects generated from the Three-way ANOVA model. To avoid both type I (due to multiple comparisons) and type II (due to too-stringent thresholds) errors, for simplicity, the statistical significance set for the *post-hoc* analyses was height-thresholded at *p* < 0.05 (*T*-tests, one-tailed) and survived peak-level FWE *p* < 0.004 in the clusters of interest.

The dissociation in the early processing was tested within the 100–200 ms space (scalp × time) for the Level of Attractiveness effect (height-thresholded at *p* < 0.001) outside a mask showing the Level of Expression effect (height-thresholded at *p* < 0.05). In other words, the effect of Expression was removed when testing for the effect of Attractiveness. The same procedure was repeated for testing Level of Expression effect outside a mask showing Level of Attractiveness effect. A lenient threshold of *p* < 0.05 was adopted, which is common in testing the dissociation effect in other neuroimaging studies (Pochon et al., 2002; Voon et al., 2014).

### ERP source reconstruction analysis

The sources of ERP signals were reconstructed and analyzed by the group inversion (imaging method) module in the SPM12 software. The electrode positions over the scalp of each participant were aligned with the standard template devised in SPM12. The ERP data of each event for each participant was inverted using the multiple sparse priors approach (Friston et al., 2008). The process modeled the scalp EEG signals as the activities resulting from numbers of dipolar sources distributed over the cortical sheet with fixed locations and orientations but varied intensities across the participants (Litvak and Friston, 2008). The intensity of the identified sources was converted into the brightness of a 3D image per task event and time window of interest for each participant. The 3D images were spatially smoothed by an 8-mm FWHM and then overlaid onto a standard MNI (Montreal Neurological Institute) brain template. The images derived in the same time window were tested by a Three-way ANOVA model similar to that used for the scalp ERP analyses. Significant main and interaction effects were reported. The statistical significance set for the source analyses was *p* < 0.05 with cluster size > 150 voxels. A large cluster size was used to minimize false positive errors.

## Results

### Behavioral findings

The accuracy rates of the behavioral responses were shown in Supplementary Table 1. Besides the significant main effect of attractiveness and the main effect of expression under both Attraction and Emotion conditions (*Fs* > 9.553, *ps* < 0.006), the more interesting findings are that the interaction between attractiveness and expression was significant under both Attraction [*F*_(1, 19)_ = 101.761, *p* < 0.001] and Emotion [*F*_(1, 19)_ = 27.193, *p* < 0.001] conditions. *Post-hoc* analyses showed that, under the Attraction condition, the accuracy rates of happy expression were significantly higher than those of sad expression [*t*_(19)_ = 12.155, *p* < 0.001] when presenting attractive faces, whereas the accuracy rates of sad expression were significantly higher than those of happy expression [*t*_(19)_ = 6.720, *p* < 0.001] when showing unattractive faces. On the other hand, under the Emotion condition, the accuracy rates of attractive faces were not significantly different from those of unattractive faces [*t*_(19)_ = −1.955, *p* = 0.130] when the expression was happy, whereas the accuracy rates of unattractive faces were significantly higher than those of attractive faces [*t*_(19)_ = 9.308, *p* < 0.001] when the expression was sad. The findings suggested that the judgment of attractiveness (expression) is modulated by facial expression (attractiveness) even when the latter facial characteristic is of no interest.

### ERP findings

The statistical results are shown in Tables 1, 2. The Condition effect was found to be significant only in within the 160–200 ms period, in a cluster in the left occipital region where Attraction condition was associated with more positive amplitudes than the Emotion condition.

**Table 1:** **SPM ANOVA results on the ERP amplitudes**.

***t*-begin**	***t*-peak**	***t*-end**	**Cluster**	***Z***	**Channel**	**Area**
**MAIN EFFECT OF LEVEL OF ATTRACTIVENESS**						
150^a^	175	200	827	4.958	20	L OT
155^a^	190	200	476	4.939	99	R OT
220	240	260	582	4.461	20	L OT
510	535	610	412	4.221	66	M CP
825	845	900	430	3.833	120	R T
**MAIN EFFECT OF LEVEL OF EXPRESSION**						
150	190	305	2349	6.622	72	M P
365	580	780	12713	6.345	75	M CP
430	570	680	2330	5.114	123	R F
150	165	205	545	4.627	52	M C
**INTERACTION: LEVEL OF ATTRACTIVENESS** × **LEVEL OF EXPRESSION**						
450	580	725	2191	5.039	75	M CP
**MAIN EFFECT OF CONDITION**						
160^a^	180	200	308	3.874	45	L O
**INTERACTIONS INVOLVING CONDITION**						
NS						

*Height-thresholded at p < 0.001 and survived cluster- or peak-level FWE correction (p < 0.05) on the whole scalp and within the time window 80–980 ms*.

a *denotes significance detected within 100–200 ms only. The time of the beginning, peak significance and end of the cluster are represented by t-begin, t-peak and t-end, respectively. Cluster refers to the number of voxels in the cluster. Z is the Z-value. Channel denotes the channel nearest to the peak significance. Area denotes the spatial distribution on the scalp. NS, no significance; L, left; M, medial; R, right; F, frontal; C, central; CP, central-parietal; P, parietal; T, temporal; OT, occipital-temporal; O, occipital*.

**Table 2:** ***Post-hoc* analyses within the clusters showing significant effects shown in Table 1**.

	**Main effect of Level of Attractiveness**					
	**A2**	**UA2**	**UA1**	**A2**	**UA2**	**UA1**
	**150–200 ms, L OT area**			**155–200 ms, R OT area**		
A1	NaN	0.050	< 0.001^*^	NaN	0.191	< 0.001^*^
A2	–	0.312	< 0.001^*^	–	0.341^a^	< 0.001^*^
UA2	–	–	0.014	–	–	0.001^*^
	**220–260 ms, L OT area**			**825–900 ms, R T area**		
A1	0.403^a^	0.202	0.005	0.168^a^	NaN	0.001^*^
A2	–	0.012	< 0.001^*^	–	NaN	< 0.001^*^
UA2	–	–	0.114	–	–	0.001^*^
	**Main effect of Level of Expression**					
	**H2**	**S2**	**S1**	**H2**	**S2**	**S1**
	**150–205 ms, M C area**			**150–305 ms, M P area**		
H1	0.001^a^^*^	< 0.001^a^^*^	< 0.001^a^^*^	< 0.001^*^	< 0.001^*^	< 0.001^*^
H2	–	0.220^a^	0.047^a^	–	NaN	0.013
S2	–	–	0.136^a^	–	–	0.140
	**430–680 ms, R F area**			**365–450 ms, M CP area**		
H1	0.096	0.009^a^	0.007^a^	0.011	< 0.001^*^	< 0.001^*^
H2	–	< 0.001^a^^*^	< 0.001^a^^*^	–	< 0.001^*^	0.007
S2	–	–	0.025	–	–	0.102^a^
	**725–780 ms, M CP area**					
H1	0.016^a^	0.051	0.003^*^			
H2	–	< 0.001^*^	< 0.001^*^			
S2	–	–	0.344			
	**Level of Attractiveness** × **Level of Expression, 450–725 ms, M CP area**					
	**A2**	**UA2**	**UA1**	**A2**	**UA2**	**UA1**
	**H1**			**H2**		
A1	0.740	0.002^*^	0.005	0.805	0.547	0.137
A2	–	0.005	0.077	–	0.316	0.109
UA2	–	–	0.587^a^	–	–	0.303
	**S2**			**S1**		
A1	0.306	0.001^*^	0.562	0.408	0.619^a^	0.001^a^^*^
A2	–	0.026	0.041^a^	–	0.093^a^	< 0.001^a^^*^
UA2	–	–	0.006^a^	–	–	0.001^a^^*^

Values shown in the table were p-values of T-tests (peak-level FWE corrected within the cluster of interest defined in ANOVA) between variables in rows and variables in columns. The p-values with (without) a superscript character (

a ) refer to variables in columns that are smaller (greater) than variables in rows. An asterisk (

* *) denotes that the p-value survived a Bonferroni correction for multiple comparisons in the post-hoc analyses. A1, attractive; A2, less attractive; UA2, less unattractive; UA1, unattractive; H1, happy; H2, less happy; S2, less sad; S1, sad*.

**Figure 2:**
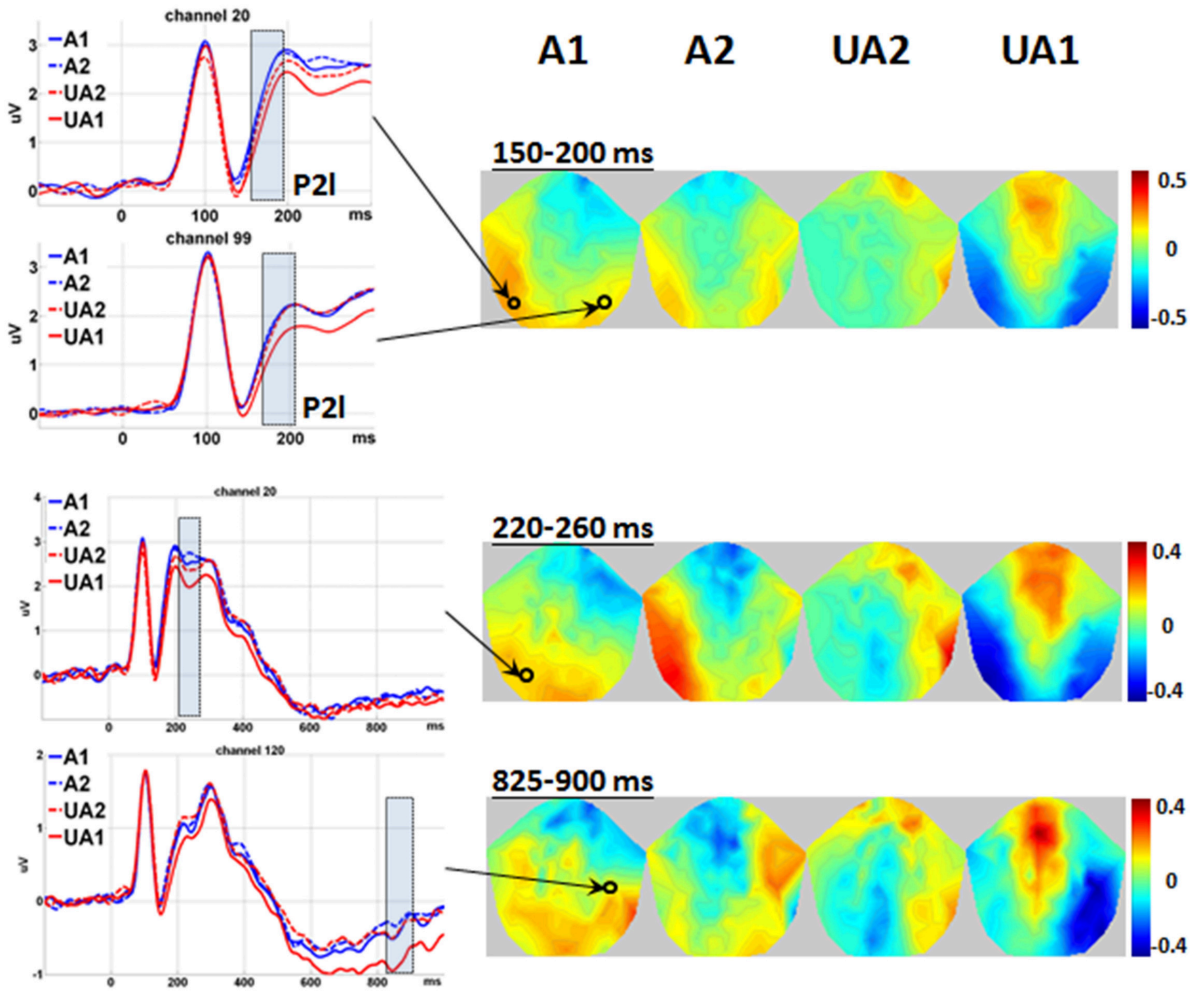
**Main effect of level of attractiveness**. Waveforms are shown in the representative channels 20 (left occipital-temporal), 99 (right occipital-temporal), and 120 (right temporal). The waveforms of P2l are only shown from −100 to 300 ms to clarify the details of each level of Level of Attractiveness. The 2D topographies are shown for the amplitudes averaged within the intervals 150–200, 220–260, and 825–900 ms, respectively. To clarify the difference between different levels of Attractiveness, mean amplitudes averaged across all conditions were removed from the 2D topographies. The shadowed bars represent the time windows' detecting significance, the small circles locate the representative channels, and the color bar denotes the range of amplitudes (μV). A1, attractive; A2, less attractive; UA2, less unattractive; UA1, unattractive.

The waveforms and 2D topographies for the Level of Attractiveness effect are presented in Figure 2. The Level of Attractiveness effect was found to be significant in three voxel clusters within the 80–980 ms time window. The first cluster was in the left occipito-temporal region within the 220–260 ms period. The UA1 faces elicited more negative-going amplitudes than did the A2 faces. The second cluster was in the medial centro-parietal region within the 510–610 ms period. The results of this cluster will be elaborated later under the results of the interaction between Level of Attractiveness and Level of Expression. The third cluster was at the right temporal region within the 825–900 ms period. *Post-hoc* analyses showed that UA1 faces elicited more negative-going amplitudes than any other faces.

Within the special time window of 100–200 ms, the Level of Attractiveness effect was found to be most significant in two voxel clusters: the left and right occipito-temporal regions within 150–200 ms. The effects were in fact most prominent at the rising edge of P2, and were thus defined as P2-lateral (P2l).

**Figure 3:**
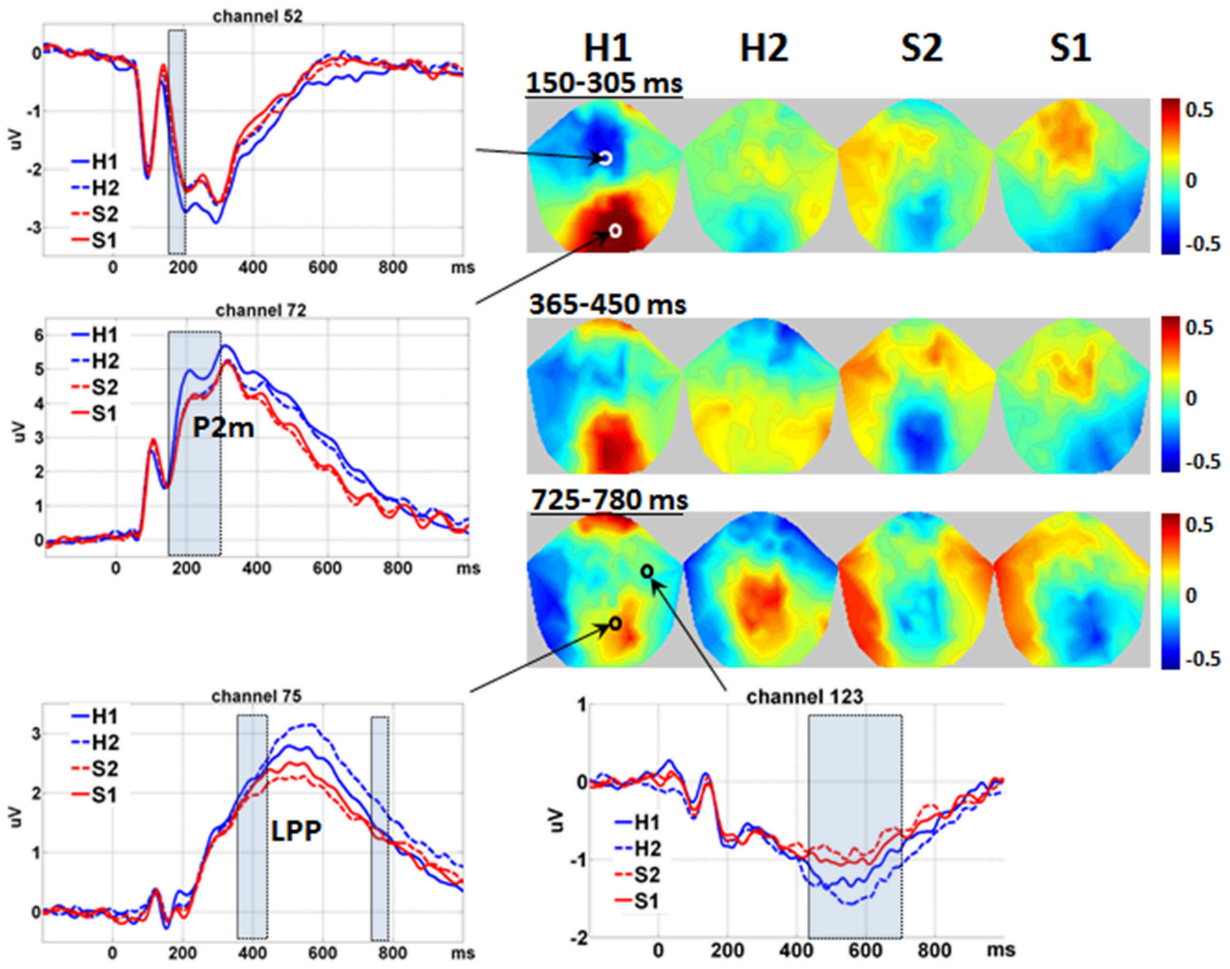
**Main effect of level of expression**. Waveforms are shown in the representative channels 52 (medial central), 72 (medial parietal), 75 (medial central-parietal), and 123 (right frontal) according to Table 1. The shadowed bars represent the time windows' detecting significance. Please notice that the interval of 450–725 ms is not covered to avoid overlap with the interaction effect between Level of Attractiveness and Level of Expression. For the same reason, the 2D topographies are shown for the amplitudes averaged within the intervals 150–305 ms (150–205 ms is not shown for simplicity), 365–450 ms and 725–780 ms, respectively. To clarify the difference between different levels of Expression, mean amplitudes averaged across all conditions were removed from the 2D topographies. The small circles locate the representative channels, and the color bar denotes the range of amplitudes (μV). H1, happy; H2, less happy; S2, less sad; S1, sad.

The waveforms and 2D topographies for Level of Expression effect are presented in Figure 3. The Level of Expression effect was also found to be significant in voxel clusters within the 80–980 ms time window. To avoid being confused by the interaction between Level of Attractiveness and Level of Expression in the 450–725 ms range and distributed at the medial central-parietal areas (which corresponded to the LPP), the cluster showing a significant Level of Expression effect within the 365–780 ms period was further separated into two clusters covering 365–450 ms and 725–780 ms periods. The first cluster was in the medial centro-parietal region, which showed a positive-going deflection at the 365–450 ms period. This suggested the elicitation of the LPP, of which H1 faces were more positive-going than either S1 or S2 faces, and H2 faces were more positive-going than the S2 faces. Within the cluster at the 725–780 ms interval, H1 faces were more positive-going than S1 faces, and H2 faces were more positive-going than either S1 or S2 faces. Another cluster showing a significant Level of Expression effect was found in the frontal region, which showed a negative-going deflection within the 430–680 ms range. The H2 faces were more negative-going than either S1 or S2 faces.

Within the special time window of 100–200 ms, the Level of Expression effect was further found to be significant in two voxel clusters. For the first cluster, it was in the medial parietal region, eliciting a positive-going deflection around 190 ms. This corresponded closely to the elicitation of the P2 component, and was defined as P2-medial (P2m) to differentiate it from the P2l associated with attractiveness. The H1 faces were found to have more positive-going P2m than all other faces. Its negative-going counterpart was detected in the medial central region. Similarly, all other faces were more negative-going than the H1 faces. It is noteworthy that the effects of Level of Expression and Level of Attractiveness had similar times of onset (i.e., 150 ms) but different spatial distributions. The Level of Expression was found to be most significant in the medial parietal region (for P2m), and the Level of Attractiveness was most significant in the bilateral occipito-temporal regions (for P2l).

**Figure 4:**
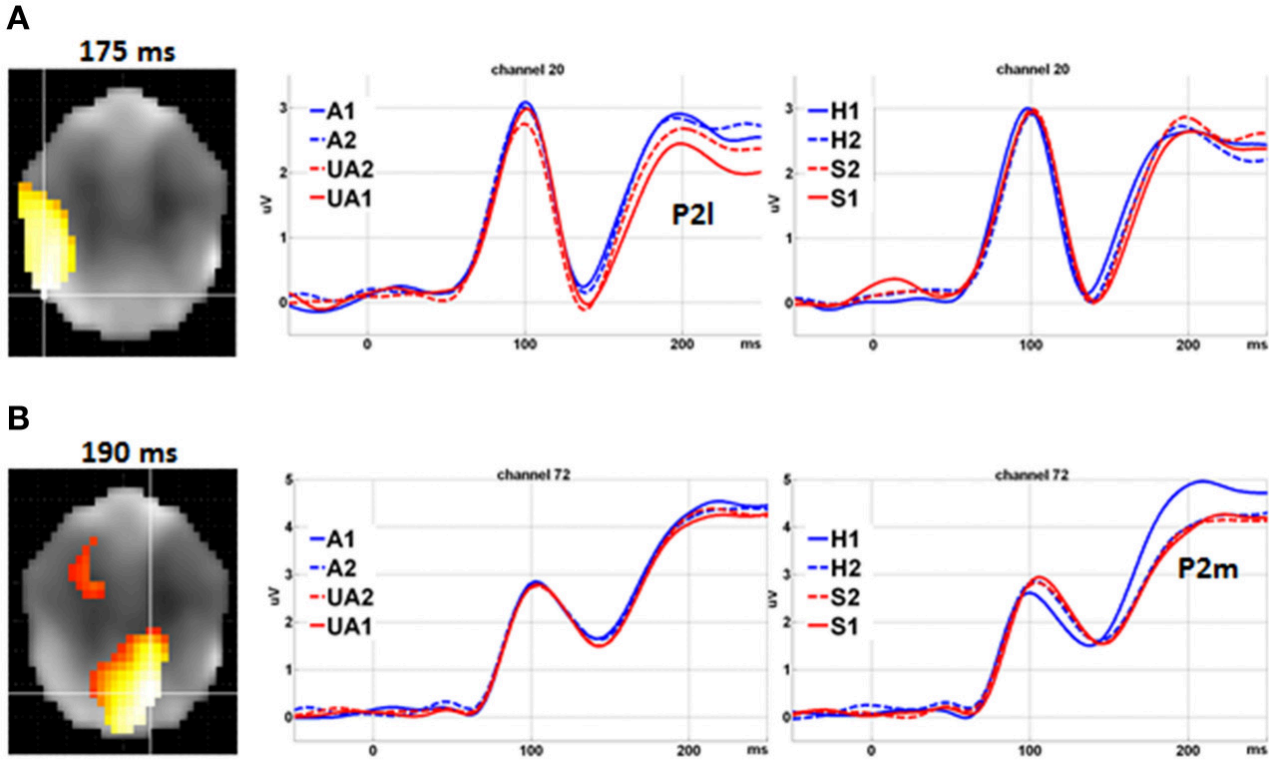
**Double dissociation between level of attractiveness and level of expression**. **(A)** Main effect of Level of Attractiveness after excluding the effects of Level of Expression. Waveforms in channel 20 at left occipital-temporal sites showed clear differences for different levels of Attractiveness (reflected by P2l) but not for different levels of Expression. **(B)** Main effect of Level of Expression after excluding the effects of Level of Attractiveness. Waveforms in channel 72 at medial parietal sites showed clear differences among different levels of Expression (reflected by P2m) but not for different levels of Attractiveness. A1, attractive; A2, less attractive; UA2, less unattractive; UA1, unattractive; H1, happy; H2, less happy; S2, less sad; S1, sad.

Further analysis supported the double dissociation between the Level of Attractiveness and Level of Expression effects within the 100–200 ms time window (Figure 4). The Level of Attractiveness effect was found to peak in the left occipital-temporal sites at 175 ms (*Z* = 4.928, cluster size = 492 voxels) after excluding the Level of Expression effect. In contrast, after excluding the Level of Attractiveness effect, the Level of Expression effect was found to peak in the medial parietal sites at 190 ms (*Z* = 7.151, cluster size = 710 voxels) and in the medial fronto-central sites at 165 ms (*Z* = 4.848, cluster size = 176 voxels).

**Figure 5:**
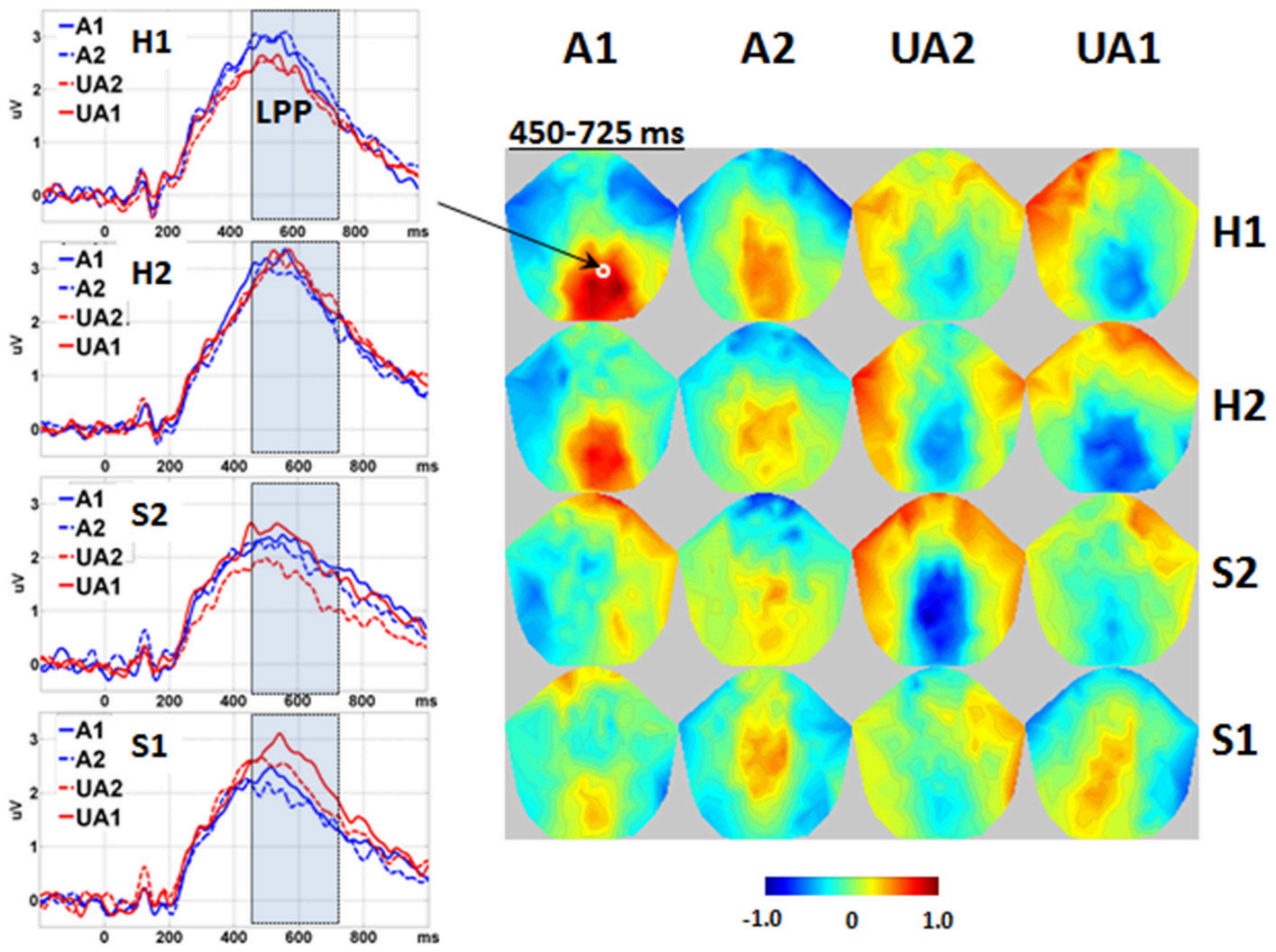
**Interaction between level of attractiveness and level of expression**. Waveforms are shown in the representative channel 75 (medial central-parietal). The 2D topographies are shown for the amplitudes averaged within the interval 450–725 ms. To clarify the difference between conditions, mean amplitudes averaged across all conditions were removed from the 2D topographies. The shadowed bars represent the time windows' detecting significance, the small circles locate the representative channels, and the color bar denotes the range of amplitudes (μV). A1, attractive; A2, less attractive; UA2, less unattractive; UA1, unattractive; H1, happy; H2, less happy; S2, less sad; S1, sad.

Significant interactions were found between the Level of Attractiveness and Level of Expression effects in the voxel cluster in the medial centro-parietal region within the 450–725 ms time window (Figure 5). These corresponded to the LPP, in which A1 faces were found to elicit more positive amplitudes than the UA2 faces when the expression was either H1 or S2. In contrast, the UA1 faces elicited more positive amplitudes than all other faces when the expression was S1.

### Source analysis findings

**Figure 6:**
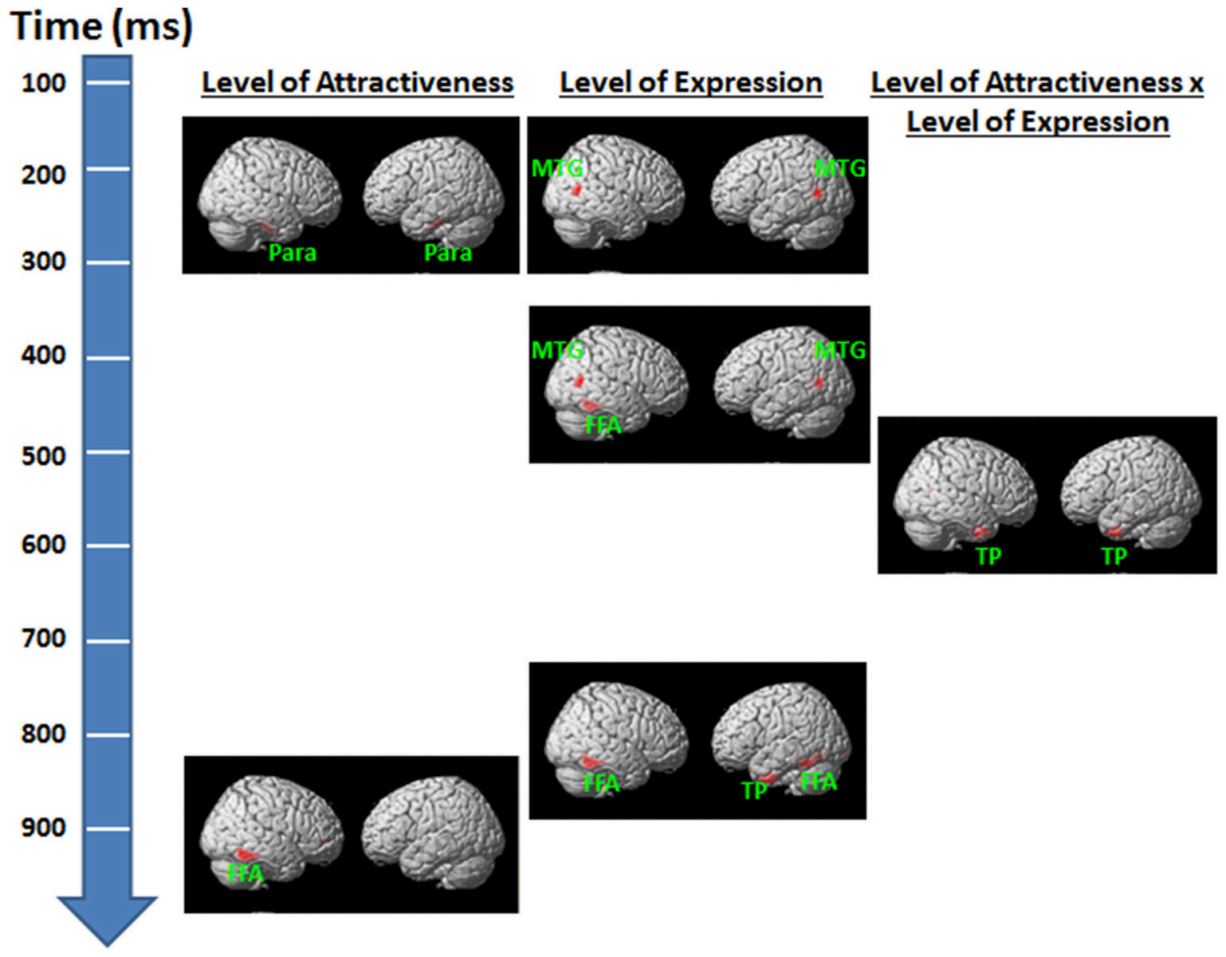
**Source reconstruction results for the effects of level of attractiveness and level of expression and their interaction in each time window of interest**. For observation purposes, results are height-thresholded at *p* < 0.05 with cluster size > 150 voxels. FFA, fusiform area; MTG, middle temporal gyrus; Para, parahippocampal gyrus; TP, temporal pole.

For the Level of Attractiveness effect, significant activities were detected in sites that coincided with locations in the bilateral parahippocampal gyri within the 150–200 ms and 220–260 ms periods (Figure 6). Another significant site was identified in the right fusiform gyrus within the 825–900 ms period. For the Level of Expression effect, significant activities were detected at the site that coincided with the bilateral middle temporal gyri within the 150–305 ms and 365–450 ms periods. A few more sites were identified, with one detected in the right fusiform gyrus within the 365–450 ms period and another in the bilateral fusiform gyri and the left temporal gyri within the 725–780 ms period. For the interaction between Level of Attractiveness and Level of Expression, significant activities were detected in the site that coincided with the bilateral temporal poles within the 450–725 ms period.

## Discussion

The results suggested that the Level of Attractiveness and Level of Expression were reflected by two early ERP components, P2l and P2m, respectively. Significant interaction effects were found in a late ERP component, LPP. These findings supported the hypothesis that facial attractiveness and expression are likely to be processed separately in the early phase and then integrated for processing in the late phase. To our knowledge, this study is the first to dissociate the neural processes of perceiving attractiveness and emotional expression, filling the gap in the classical model of face perception (Bruce and Young, 1986; Haxby et al., 2000).

### Early processing before 350 ms

No interaction effects were revealed on the Level of Attractiveness and Level of Expression in the early stage of face perception. This result suggested that facial attractiveness and facial expression are likely to be processed independently soon after the subjects viewed the face stimuli. This was supported by the different scalp and temporal distributions and amplitudes elicited by the different levels for the attractiveness and expression conditions. First, the amplitudes of the Level of Attractiveness effect were the most significant at the bilateral occipital-temporal sites (P2l), while the Level of Expression effect reached its peak significance at the medial parietal (P2m) and medial central sites. Second, although both effects began from 150 ms after the face onset, the Level of Attractiveness effect ended at 200 ms while the Level of Expression effect ended at 305 ms. Third, significant P2l deflection was observed between the faces with the most negative valence in Level of Attractiveness (i.e., UA1 or unattractive faces) and all other faces, whereas significant P2m deflection was detected between the faces with the most positive valence in Level of Expression (i.e., H1 or happy faces) and all other faces. Using the “exclusive mask” method, significant Level of Attractiveness effects at the left occipital-temporal sites (P2l) were identified after excluding the Level of Expression effect. In contrast, significant Level of Expression effects were identified at the medial parietal and medial frontal-central sites (P2m) after excluding the Level of Attractiveness effect. These converging findings showed a double dissociation between the two effects within the 100–200 ms range. They support our hypothesis that processing of facial attractiveness and facial emotional expression are distinct during the early stage of face perception. Given that P2 has been supposed to reflect the comparison between sensory input and stored memory (Luck and Hillyard, 1994) and initial “attention capture” of (physically) distinctive faces (van Hooff et al., 2011), our findings suggest that faces at the early stage of processing are compared with stored prototypes and/or are allocated with attentions in the dimensions of attractiveness and expression in parallel. This idea is consistent with the opinion that varied aspects of the face may be extracted in parallel to build a multi-dimensional space (Freeman et al., 2010). Previous studies reported that P2 was modified by facial attractiveness (Halit et al., 2000; van Hooff et al., 2011; Zhang and Deng, 2012) or facial expression (Spreckelmeyer et al., 2006; Ofsson and Polich, 2007). Their findings are different from those revealed in this study. All of these studies did not concurrently manipulate both effects, which perhaps introduced confounding effects in their study design. In our study, facial attractiveness and expression were manipulated in the experimental task. The difference in the task designs is likely to contribute to the difference in interpretation of the functionality of P2.

The results of the source reconstruction offer further insight into the neural processing of facial attractiveness and emotional expression. Bilateral parahippocampal gyri were identified as the key neural substrate associated with the attractiveness effects. This contrasted to the bilateral middle temporal gyri associated with the facial expression effects. The parahippocampal gyri are adjacent to the fusiform gyri which have been found to respond stronger to attractive (vs. neutral) faces (Chatterjee et al., 2009). Given that our source results were only derived from the scalp ERP data and might be biased in spatial localization, the findings in parahippocampal gyri could have been originated from the fusiform gyri. On the other hand, the middle temporal gyrus has often been reported in studies on emotion (Sabatinelli et al., 2011), although there is no direct evidence supporting the relationship between this area and P2. These results suggested that the brain areas involved are different for the two types of face information in the early stage of face perception.

It is noteworthy that besides P2l and P2m, this study identified a third component elicited within the 220–260 ms time-window at the left occipital-temporal sites associating with the Level of Attractiveness but not the Level of Expression effect. The results indicated that the less attractive faces (A2) elicited more positive amplitudes than the unattractive faces (UA1). Burkhardt et al. (2010) reported a positive deflection peaked at around 250 ms, identified as P250, at the bilateral temporal-occipital sites. The amplitude was found decreased with the increase in the amount of distortion (compressive or expansive) of a face. Burkhardt et al.'s study has two implications. First, the component associated with the facial attractiveness effects could be P250. Second, unattractive faces could have been perceived by subjects as distorted representation of average faces, of which the latter were regarded as norm or mental (Langlois and Roggman, 1990; Rhodes et al., 1999). Another possibility of this third component associating with facial attractiveness is an early posterior negativity (EPN) elicited within the 220–260 ms. Previous studies suggested that EPN was associated with visual attention to emotional stimuli (Junghöfer et al., 2001; Schupp et al., 2007). However, other studies reported more-negative EPN for attractive than unattractive faces (Werheid et al., 2007) or for highly attractive than for medium- or low-attractive faces (Marzi and Viggiano, 2010). These findings are in contrary to results of this study. Future studies are called for to verify the association of P250 or EPN with early processing of the facial attractiveness effects.

### Late processing after 350 ms

Significant interactions were revealed between the Level of Attractiveness and Level of Expression effects. Within the 450–725 ms range, the unattractive sad faces (UA1 in S1 condition) were found to elicit more positive LPP than all the other sad faces; on the contrary, attractive happy faces (A1 in H1 condition) also elicited more positive LPP than less unattractive happy faces. It appears that the LPP was modulated and enhanced by both the attractiveness and expression effects along the same direction of valence. The strongest effects were found in negative valences, i.e., unattractive faces with sad expression. Previous studies suggested that LPP reflects late neural process of allocating attentional resources on stimuli of high intrinsic motivational properties (Johnston and Oliver-Rodriguez, 1997; Cuthbert et al., 2000; Werheid et al., 2007; Foti et al., 2009). In other words, late processing of face perception is likely to tap on an increase in attentional resource intensified by unattractive faces with sad expressions. This observation coincides with the “negativity bias” in which unpleasant stimuli were found to produce stronger emotional effects than pleasant stimuli (Crawford and Cacioppo, 2002). In real life situation, people would readily attend to smiling face of a pretty celebrity which can be easily forgotten. In contrast, people would be hesitated to look at sad face of an unfortunate victim which is vividly remembered for a prolonged period of time.

Some previous studies have detected U-shaped pattern of LPP for the effect of attractiveness (Schacht et al., 2008; Marzi and Viggiano, 2010). That is, larger LPP is elicited by either attractive or unattractive faces than faces of medium attractiveness. Here, we also found in some cases that faces with extreme rating on attractiveness elicited larger LPP than those with relatively neutral ratings. In another words, when the expression was either happy (H1) or less sad (S2), attractive faces (A1) elicited larger LPP than relatively more neutral faces, i.e., less unattractive faces (UA2). The findings suggested that the U-shaped pattern of LPP is modulated by both attractiveness and expression.

It should be noted that an interval showing significant effect of Level of Expression (365–450 ms) was found just before the interval showing the abovementioned interaction effect (450–725 ms). Larger LPP (rising edge) was found to be elicited more by happy (H1) than by less sad (S2) or sad (S1) faces, and more by less happy (H2) than by less sad (S2) faces. This finding suggested that positive and negative expressions would have been discriminated prior to interacting with attractiveness. This processing could have facilitated the integrative processing of attractiveness and expression during the 450–725 ms time-window.

The results of source analyses indicated that the right fusiform gyrus was identified to associate with the significant LPP amplitudes elicited by the facial expression effect within the 365–450 ms. This result is consistent with previous findings that the fusiform gyrus mediated perception of human faces (Haxby et al., 2002; Gobbini and Haxby, 2007), particularly for faces with emotional (rather than neutral) expressions (Sabatinelli et al., 2011). On the other hand, the bilateral temporal poles were identified to associate with the significant LPP amplitudes elicited by both the facial attractiveness and facial expression effects. The temporal poles were found to mediate binding of complex perceptual inputs to visceral emotional responses (Olson et al., 2007). The source analyses results further corroborate the finding that the late process of LPP within the 450–725 ms range reflects complex attention allocation and/or appraisal processing integrating the facial attractiveness and emotion effects.

We found that the interval showing significant interaction between Level of Attractiveness and Level of Expression (450–725 ms) was located within a long time window showing main effect of level of Expression (365–780 ms). Similar to the main effect of Level of Expression found at the rising edge of LPP (365–450 ms), larger LPP (descending edge, 725–780 ms) was elicited by happy (H1) than by sad (S1) faces, and more by less happy (H2) than by less sad (S2) faces or sad faces. In line with the thought that LPP reflects allocation of attention to stimuli of high intrinsic motivational properties (Werheid et al., 2007; Foti et al., 2009), our findings suggested that facial expression changes the attention allocated on the faces, and this modulation further interacts with the processing of facial attractiveness.

We also found, on the other hand, a significant main effect of Level of Attractiveness 825-900 ms. Previous ERP studies on LPP findings of facial attractiveness are often within the time window between 200 and 700 ms (Werheid et al., 2007; Marzi and Viggiano, 2010; van Hooff et al., 2011; Zhang and Deng, 2012). Few studies on attractiveness have reported the findings beyond 800 ms. Thus, the cognitive processes associated with this late component are still unclear. A previous study by Foti et al. (2009) showed that the LPP appears to include three positivities peaking at 353, 841, and 1595 ms, suggesting that LPP consists of several subcomponents. However, the neural processes reflected by the three subcomponents are still unknown. The findings by both us and other teams convergently suggest that our understanding of the late ERP components is still limited. Future studies should investigate the cognitive processes associated with the late components.

The behavioral responses showed dramatic changes of the classification of attractiveness (expression) when the Level of Expression (Attractiveness) varied. The response patterns may reflect the behavioral outputs of the interactions between Level of Attractiveness and Level of Expression. In this study, we fabricated the face stimuli in the way that the variation of Level of Attractiveness and the variation of Level of Expression are independent from each other. The selected face stimuli thus allow us to investigate the neural correlates of either facial attractiveness or expression without being confounded by participants' subjective judgment. Future studies should further address the question that how the judgment of either attractiveness or expression is influenced by task requirement.

Findings in this study are consistent with studies on romantic love. For example, enhanced LPP was found to be elicited by beloved-related (vs. friend-related) face images (Langeslag et al., 2007) and words/phrases (Langeslag et al., 2015), suggesting that attention is enhanced for beloved-related information. A recent study using magnetoencephalography (MEG) also reported results comparable to the LPP effect (Tiedt et al., 2014). In line with our findings and the aforementioned discussions, facial attractiveness and facial expression may affect the processing of beloved-related information by influencing the attention given to it. This effect may be reflected by late-latency components, including LPP. This hypothesis should be tested in future studies on the neural processing of romantic love.

## Conclusion

This study delineated the time course of neural processing for perceiving facial attractiveness and facial expression. In early processing, facial attractiveness (reflected by P2l), and facial expression (reflected by P2m) are likely to be processed separately for discrimination between stimuli during the early stage of face perception. In later processing, more attentional resources (reflected by LPP) would be allocated to the faces with the most positive or most negative valences in either attractiveness or expression. Finally, the faces are processed separately (reflected by slow waves). These findings contribute to advancing the theoretical model of face perception.

### Conflict of interest statement

The authors declare that the research was conducted in the absence of any commercial or financial relationships that could be construed as a potential conflict of interest.

## References

[B1] Bruce V. Young A. (1986). Understanding face recognition. Br. J. Psychol. 77(Pt 3), 305–327. 10.1111/j.2044-8295.1986.tb02199.x 3756376

[B2] Burkhardt A. Blaha L. M. Jurs B. S. Rhodes G. Jeffery L. Wyatte D.. (2010). Adaptation modulates the electrophysiological substrates of perceived facial distortion: support for opponent coding. Neuropsychologia 48, 3743–3756. 10.1016/j.neuropsychologia.2010.08.016 20736026

[B3] Chatterjee A. Thomas A. Smith S. E. Aguirre G. K. (2009). The neural response to facial attractiveness. Neuropsychology 23, 135–143. 10.1037/a0014430 19254086

[B4] Crawford L. E. Cacioppo J. T. (2002). Learning where to look for danger: integrating affective and spatial information. Psychol. Sci. 13, 449–453. 10.1111/1467-9280.00479 12219812

[B5] Cuthbert B. N. Schupp H. T. Bradley M. M. Birbaumer N. Lang P. J. (2000). Brain potentials in affective picture processing: covariation with autonomic arousal and affective report. Biol. Psychol. 52, 95–111. 10.1016/S0301-0511(99)00044-7 10699350

[B6] Dong G. Wu H. Lu Q. (2010). Attempting to hide our real thoughts: electrophysiological evidence from truthful and deceptive responses during evaluation. Neurosci. Lett. 479, 1–5. 10.1016/j.neulet.2010.05.014 20470861

[B7] Eimer M. Holmes A. (2007). Event-related brain potential correlates of emotional face processing. Neuropsychologia 45, 15–31. 10.1016/j.neuropsychologia.2006.04.022 16797614 PMC2383989

[B8] Ekman P. (2003). Emotions Revealed: Recognizing Faces and Feelings to Improve Communication and Emotional Life, 1st Edn. New York, NY: Times Books.

[B9] Fisher H. Aron A. Brown L. L. (2005). Romantic love: an fMRI study of a neural mechanism for mate choice. J. Comp. Neurol. 493, 58–62. 10.1002/cne.20772 16255001

[B10] Foti D. Hajcak G. Dien J. (2009). Differentiating neural responses to emotional pictures: evidence from temporal-spatial PCA. Psychophysiology 46, 521–530. 10.1111/j.1469-8986.2009.00796.x 19496228

[B11] Freeman J. Ambady N. Holcomb P. J. (2010). The face-sensitive N170 encodes social category information. Neuroreport 21, 24–28. 10.1097/WNR.0b013e3283320d54 19864961 PMC3576572

[B12] Friston K. Harrison L. Daunizeau J. Kiebel S. Phillips C. Trujillo-Barreto N.. (2008). Multiple sparse priors for the M/EEG inverse problem. Neuroimage 39, 1104–1120. 10.1016/j.neuroimage.2007.09.048 17997111

[B13] Fusar-Poli P. Placentino A. Carletti F. Landi P. Allen P. Surguladze S.. (2009). Functional atlas of emotional faces processing: a voxel-based meta-analysis of 105 functional magnetic resonance imaging studies. J. Psychiatr. Neurosci. 34, 418–432. 19949718 PMC2783433

[B14] Gobbini M. I. Haxby J. V. (2007). Neural systems for recognition of familiar faces. Neuropsychologia 45, 32–41. 10.1016/j.neuropsychologia.2006.04.015 16797608

[B15] Gratton G. Coles M. G. Donchin E. (1983). A new method for off-line removal of ocular artifact. Electroencephalogr. Clin. Neurophysiol. 55, 468–484. 10.1016/0013-4694(83)90135-9 6187540

[B16] Halit H. de Haan M. Johnson M. H. (2000). Modulation of event-related potentials by prototypical and atypical faces. Neuroreport 11, 1871–1875. 10.1097/00001756-200006260-00014 10884035

[B17] Haxby J. V. Hoffman E. A. Gobbini M. I. (2000). The distributed human neural system for face perception. Trends Cogn. Sci. 4, 223–233. 10.1016/S1364-6613(00)01482-0 10827445

[B18] Haxby J. V. Hoffman E. A. Gobbini M. I. (2002). Human neural systems for face recognition and social communication. Biol. Psychiatry 51, 59–67. 10.1016/S0006-3223(01)01330-0 11801231

[B19] Ishai A. (2007). Sex, beauty and the orbitofrontal cortex. Int. J. Psychophysiol. 63, 181–185. 10.1016/j.ijpsycho.2006.03.010 16759727

[B20] Johnston V. S. Oliver-Rodriguez J. C. (1997). Facial beauty and the late positive component of event-related potentials. J. Sex Res. 34, 188–198. 10.1080/00224499709551884

[B21] Junghöfer M. Bradley M. M. Elbert T. R. Lang P. J. (2001). Fleeting images: a new look at early emotion discrimination. Psychophysiology 38, 175–178. 10.1111/1469-8986.3820175 11347862

[B22] Langeslag S. J. Olivier J. R. Köhlen M. E. Nijs I. M. Van Strien J. W. (2015). Increased attention and memory for beloved-related information during infatuation: behavioral and electrophysiological data. Soc. Cogn. Affect. Neurosci. 10, 136–144. 10.1093/scan/nsu034 24526182 PMC4994849

[B23] Langeslag S. J. E. Jansma B. A. Franken I. H. A. Van Strien J. W. (2007). Event-related potential responses to love-related facial stimuli. Biol. Psychol. 76, 109–115. 10.1016/j.biopsycho.2007.06.007 17681417

[B24] Langlois J. H. Roggman L. A. (1990). Attractive faces are only average. Psychol. Sci. 1, 115–121. 10.1111/j.1467-9280.1990.tb00079.x

[B25] Little A. C. Jones B. C. DeBruine L. M. (2011). Facial attractiveness: evolutionary based research. Philos. Trans. R. Soc. B 366, 1638–1659. 10.1098/rstb.2010.0404 21536551 PMC3130383

[B26] Little A. C. (2014). Facial attractiveness. Wires Cogn. Sci. 5, 621–634. 10.1002/wcs.1316 26308869

[B27] Litvak V. Friston K. (2008). Electromagnetic source reconstruction for group studies. Neuroimage 42, 1490–1498. 10.1016/j.neuroimage.2008.06.022 18639641 PMC2581487

[B28] Litvak V. Mattout J. Kiebel S. Phillips C. Henson R. Kilner J.. (2011). EEG and MEG data analysis in SPM8. Comput. Intell. Neurosci. 2011, 852961. 10.1155/2011/852961 21437221 PMC3061292

[B29] Liu X. F. Liao Y. Zhou L. P. Sun G. Li M. Zhao L. (2013). Mapping the time course of the positive classification advantage: an ERP study. Cogn. Affect. Behav. Neurosci. 13, 491–500. 10.3758/s13415-013-0158-6 23504806

[B30] Luck S. J. Hillyard S. A. (1994). Electrophysiological correlates of feature analysis during visual-search. Psychophysiology 31, 291–308. 10.1111/j.1469-8986.1994.tb02218.x 8008793

[B31] Luo W. Feng W. He W. Wang N. Y. Luo Y. J. (2010). Three stages of facial expression processing: ERP study with rapid serial visual presentation. Neuroimage 49, 1857–1867. 10.1016/j.neuroimage.2009.09.018 19770052 PMC3794431

[B32] Main J. C. DeBruine L. M. Little A. C. Jones B. C. (2010). Interactions among the effects of head orientation, emotional expression, and physical attractiveness on face preferences. Perception 39, 62–71. 10.1068/p6503 20301847

[B33] Marzi T. Viggiano M. P. (2010). When memory meets beauty: insights from event-related potentials. Biol. Psychol. 84, 192–205. 10.1016/j.biopsycho.2010.01.013 20109520

[B34] O'Doherty J. Winston J. Critchley H. Perrett D. Burt D. M. Dolan R. J. (2003). Beauty in a smile: the role of medial orbitofrontal cortex in facial attractiveness. Neuropsychologia 41, 147–155. 10.1016/S0028-3932(02)00145-8 12459213

[B35] Ofsson J. K. Polich J. (2007). Affective visual event-related potentials: arousal, repetition, and time-on-task. Biol. Psychol. 75, 101–108. 10.1016/j.biopsycho.2006.12.006 17275979 PMC1885422

[B36] Oldfield R. C. (1971). The assessment and analysis of handedness: the Edinburgh inventory. Neuropsychologia 9, 97–113. 10.1016/0028-3932(71)90067-4 5146491

[B37] Olofsson J. K. Nordin S. Sequeira H. Polich J. (2008). Affective picture processing: an integrative review of ERP findings. Biol. Psychol. 77, 247–265. 10.1016/j.biopsycho.2007.11.006 18164800 PMC2443061

[B38] Olson I. R. Ploaker A. Ezzyat Y. (2007). The Enigmatic temporal pole: a review of findings on social and emotional processing. Brain 130, 1718–1731. 10.1093/brain/awm052 17392317

[B39] Otta E. Abrosio F. F. E. Hoshino R. L. (1996). Reading a smiling face: messages conveyed by various forms of smiling. Percept. Mot. Skill. 82, 1111–1121. 10.2466/pms.1996.82.3c.1111 8823879

[B40] Pochon J. B. Levy R. Fossati P. Lehericy S. Poline J. B. Pillon B.. (2002). The neural system that bridges reward and cognition in humans: an fMRI study. Proc. Natl. Acad. Sci. U.S.A. 99, 5669–5674. 10.1073/pnas.082111099 11960021 PMC122829

[B41] Rhodes G. Sumich A. Byatt G. (1999). Are average facial configurations attractive only because of their symmetry? Psychol. Sci. 10, 52–58. 10.1111/1467-9280.00106

[B42] Sabatinelli D. Fortune E. E. Li Q. Y. Siddiqui A. Krafft C. Oliver W. T.. (2011). Emotional perception: meta-analyses of face and natural scene processing. Neuroimage 54, 2524–2533. 10.1016/j.neuroimage.2010.10.011 20951215

[B43] Schacht A. Werheid K. Sommer W. (2008). The appraisal of facial beauty is rapid but not mandatory. Cogn. Affect. Behav. Neurosci. 8, 132–142. 10.3758/CABN.8.2.132 18589504

[B44] Schupp H. T. Stockburger J. Codispoti M. Junghöfer M. Weike A. I. Hamm A. O. (2007). Selective visual attention to emotion. J. Neurosci. 27, 1082–1089. 10.1523/JNEUROSCI.3223-06.2007 17267562 PMC6673176

[B45] Spreckelmeyer K. N. Kutas M. Urbach T. P. Altenmüller E. Münte T. F. (2006). Combined perception of emotion in pictures and musical sounds. Brain Res. 1070, 160–170. 10.1016/j.brainres.2005.11.075 16403462

[B46] Sun D. Chan C. C. Lee T. M. (2012). Identification and classification of facial familiarity in directed lying: an ERP study. PLoS ONE 7:e31250. 10.1371/journal.pone.0031250 22363597 PMC3283635

[B47] Sun D. Lee T. M. Chan C. C. (2015). Unfolding the Spatial and Temporal Neural Processing of Lying about Face Familiarity. Cereb. Cortex 25, 927–936. 10.1093/cercor/bht284 24186897 PMC4379998

[B48] Tiedt H. O. Beier K. M. Lueschow A. Pauls A. Weber J. E. (2014). A different pattern of lateralised brain activity during processing of loved faces in men and women: a MEG study. Biol. Psychol. 103, 255–261. 10.1016/j.biopsycho.2014.09.014 25312880

[B49] van Hooff J. C. Crawford H. van Vugt M. (2011). The wandering mind of men: ERP evidence for gender differences in attention bias towards attractive opposite sex faces. Soc. Cogn. Affect. Neursci. 6, 477–485. 10.1093/scan/nsq066 20601424 PMC3150857

[B50] Voon V. Mole T. B. Banca P. Porter L. Morris L. Mitchell S.. (2014). Neural correlates of sexual cue reactivity in individuals with and without compulsive sexual behaviours. PLoS ONE 9:e102419. 10.1371/journal.pone.0102419 25013940 PMC4094516

[B51] Vuilleumier P. Pourtois G. (2007). Distributed and interactive brain mechanisms during emotion face perception: evidence from functional neuroimaging. Neuropsychologia 45, 174–194. 10.1016/j.neuropsychologia.2006.06.003 16854439

[B52] Werheid K. Schacht A. Sommer W. (2007). Facial attractiveness modulates early and late event-related brain potentials. Biol. Psychol. 76, 100–108. 10.1016/j.biopsycho.2007.06.008 17681418

[B53] Winston J. S. O'Doherty J. Kilner J. M. Perrett D. I. Dolan R. J. (2007). Brain systems for assessing facial attractiveness. Neuropsychologia 45, 195–206. 10.1016/j.neuropsychologia.2006.05.009 16828125

[B54] Zhang Z. Deng Z. (2012). Gender, facial attractiveness, and early and late event-related potential components. J. Integr. Neurosci. 11, 477–487. 10.1142/S0219635212500306 23351053

