# Supplementary material for: Are Happy Faces Attractive? The Roles of Early vs. Late Processing
Source: Front Psychol. 2015 Nov 30;6:1812. doi: 10.3389/fpsyg.2015.01812 (PMC4663264; doi:10.3389/fpsyg.2015.01812)
Supplement: Supplementary file 1 [file Table1.DOCX]

Supplemental Material for:

**Are Happy Faces Attractive? The Roles of Early versus Late Processing**

**Running title: Neural Correlates of Attractiveness & Expression**

Delin Sun^1,†^, Chetwyn C.H. Chan^2,†,*^, Jintu Fan^3^, Yi Wu^4^ , Tatia M.C. Lee^1,5*^

^1^ Laboratory of Neuropsychology, The University of Hong Kong, Hong Kong

^2^ Applied Cognitive Neuroscience Laboratory, The Hong Kong Polytechnic University, Hong Kong

^3^ Department of Fiber Science and Apparel Design, Connell University, United States

^4^ Department of Rehabilitation Medicine, Huashan Hospital, Fudan University, Shanghai, China

^5^ Institute of Clinical Neuropsychology, The University of Hong Kong, Hong Kong.

This file includes:

**Supplementary Methods**

Traditional analyses of ERP components

**Supplementary Tables**

Supplementary Table 1

Supplementary Table 2

Supplementary Table 3

**Traditional analyses of ERP components**

The traditional analyses of ERP data based on the time windows and channels selected *a priori* were conducted to compare with findings by SPM method. The time windows and channels of interest were selected based on both literatures and visual inspections on our data. For the P2 components, Halit (2000) investigated peak amplitudes within 188-300 ms at bilateral medial, semimedial and lateral sites. van Hooff et al. (2011) measured peak amplitudes within 120-220 ms at Pz channel. Zhang et al. (2012) recorded peak amplitudes within 150-230 ms from frontal to parietal areas and across left, medial and right sides. Their findings on P2 were most prominent at posterior sites. Observation of our data showed a significant P2 peak around 200 ms at posterior sites. We thus investigated both peak amplitudes within 170-230 ms at left (channels 18, 20 and 21), medial (channels 46, 67, 68 and 72) and right (channels 99, 100 and 118) sites.

For the LPP component, on the other hand, Marzi et al. (2010) investigated mean amplitudes in two consecutive time windows covering 300-700 ms at 15 sites from frontal to parietal areas and across left, medial and right sides. van Hooff et al. (2011) measured mean amplitudes within three consecutive time windows covering 200-650 ms at 9 sites from frontal to parietal areas and across left, medial and right sides. Zhang et al. (2012) recorded mean amplitudes within 330-500 ms at 18 sites from frontal to parietal areas and across left, medial and right sides. Their findings on LPP were distributed along the midline. Observation of our data showed a significant large positivity from 350 ms to 800 ms at medial parietal sites. We thus investigated mean amplitudes in three consecutive time windows covering 350-800 ms, i.e. 350-500 ms, 500-650 ms and 650-800 ms, at three sites along the midline, i.e. frontal (channels 55, 59, 60 and 81), central (channels 10, 50, 64 and 76) and parietal (channels 46, 67, 68 and 72).

The values extracted were entered into 2 (Condition: Attraction and Emotion) by 4 (Level of Attractiveness: A1, A2, UA2 and UA1) by 4 (Level of Expression: H1, H2, S2 and S1) by ~~2~~ 3 (Channel location: left, medial and right for P2; frontal, central and parietal for LPP) repeated measures ANOVA models. Greenhouse-Geisser method was applied whatever the assumption of sphericity was violated. Post hoc analyses with Bonferroni correction were carried out if significance was detected for the effect of interest.

The statistical *F* and *p* values were listed in Supplementary Table 1. For the P2 component, both the interaction between Channel location and Level of Expression (*F*(6,114) = 7.991, *p* < 0.001), and the interaction between Channel location and Level of Attractiveness (*F*(6,114) = 2.623, *p* = 0.043) were found significant, suggesting that the scalp distribution is different between attractiveness- and expression-associated P2 effect. Further analyses showed that, effect of Level of Attractiveness was significant at left (*F*(3,57) = 8.959, *p* < 0.001) and right (*F*(3,57) = 7.594, *p* < 0.001) sites, but not at medial sites (*F*(3,57) = 1.067, *p* = 1). By contrast, effect of Level of Expression was significant at medial (*F*(3,57) = 14.594, *p* < 0.001) sites, but not at left (*F*(3,57) = 1.200, *p* = 1) or right (*F*(3,57) = 4.034, *p* = 0.069) sites.

For the LPP component, there was a significant interaction between Channel location and Level of Expression (*F*(6,114) = 6.425, *p* < 0.001) within 350-500 ms. Further analyses showed that the effect of Level of Expression was insignificant at frontal (*F*(3,57) = 2.069, *p* = 0.369) and central (*F*(3,57) = 3.400, *p* = 0.084) sites, but was significant at parietal sites (*F*(3,57) = 10.991, *p* < 0.001).

Within 500-650 ms, there was a significant Channel location * Level of Attractiveness * Level of Expression interaction (*F*(18,342) = 2.580, *p* = 0.014). Further analyses showed that the interaction between Level of Attractiveness and Level of Expression was insignificant at frontal sites (*F*(9,171) = 1.627, *p* = 0.420), but was significant at central (*F*(9,171) = 3.043, *p* = 0.045) and parietal (*F*(9,171) = 3.792, *p* = 0.009) sites.

Within 650-800 ms, there was still a significant Channel location * Level of Attractiveness * Level of Expression interaction (*F*(18,342) = 2.074, *p* = 0.050). Further analyses showed that the interaction between Level of Attractiveness and Level of Expression was insignificant at frontal sites (*F*(9,171) = 1.911, *p* = 0.234), central (*F*(9,171) = 2.638, *p* = 0.072) and parietal (*F*(9,171) = 2.037, *p* = 0.222) sites.

Findings of traditional analyses for both P2 and LPP components were consistent with those by SPM method.

**Supplementary Table1. Statistical values of N170, P2 and LPP by the traditional ERP analyses methods.**

|  |  |  | **P2** | | **LPP** | | | | | |
| --- | --- | --- | --- | --- | --- | --- | --- | --- | --- | --- |
|  |  |  | **170-230 ms** | | **350-500 ms** | | **500-650 ms** | | **650-800 ms** | |
| **Factor** | **df1** | **df2** | ***F*** | ***p*** | ***F*** | ***p*** | ***F*** | ***p*** | ***F*** | ***p*** |
| **Loc** | 2 | 38 | 9.117 | 0.001 | 40.772 | 0.000 | 37.242 | 0.000 | 13.510 | 0.001 |
| **Con** | 1 | 19 | 5.218 | 0.034 | 1.142 | 0.299 | 0.257 | 0.618 | 0.047 | 0.831 |
| **Exp** | 3 | 57 | 7.893 | 0.002 | 9.941 | 0.000 | 10.777 | 0.000 | 6.528 | 0.003 |
| **Att** | 3 | 57 | 7.315 | 0.001 | 1.924 | 0.146 | 4.480 | 0.009 | 3.309 | 0.034 |
| **Loc * Con** | 2 | 38 | 1.480 | 0.243 | 2.966 | 0.070 | 1.652 | 0.208 | 0.863 | 0.429 |
| **Loc * Exp** | 6 | 114 | 7.991 | 0.000 | 6.425 | 0.000 | 5.544 | 0.002 | 2.498 | 0.066 |
| **Con * Exp** | 3 | 57 | 1.555 | 0.218 | 2.003 | 0.132 | 2.361 | 0.085 | 3.081 | 0.045 |
| **Loc * Con * Exp** | 6 | 114 | 0.370 | 0.829 | 1.011 | 0.399 | 1.532 | 0.212 | 1.710 | 0.173 |
| **Loc * Att** | 6 | 114 | 2.623 | 0.043 | 1.162 | 0.333 | 1.828 | 0.144 | 2.221 | 0.099 |
| **Con * Att** | 3 | 57 | 1.346 | 0.271 | 0.549 | 0.622 | 1.810 | 0.168 | 1.695 | 0.185 |
| **Loc * Con * Att** | 6 | 114 | 1.252 | 0.295 | 0.735 | 0.532 | 1.575 | 0.206 | 1.899 | 0.140 |
| **Exp * Att** | 9 | 171 | 1.230 | 0.305 | 1.370 | 0.237 | 3.353 | 0.007 | 2.386 | 0.041 |
| **Loc * Exp * Att** | 18 | 342 | 0.940 | 0.487 | 1.338 | 0.240 | 2.580 | 0.014 | 2.074 | 0.050 |
| **Con * Exp * Att** | 9 | 171 | 2.172 | 0.057 | 1.605 | 0.163 | 1.218 | 0.307 | 0.530 | 0.714 |
| **Loc * Con * Exp * Att** | 18 | 342 | 0.844 | 0.567 | 1.462 | 0.186 | 1.255 | 0.281 | 1.406 | 0.216 |

Note: F and p values were according to the Greenhouse-Geisser method whatever the assumption of sphericity was violated; while df1 and df2 were based on assumed sphericity. Loc = Channel location, Con = Condition, Exp = Level of Expression, Att = Level of Attractiveness.

**Supplementary Table 2. Accuracy rates of behavioral responses.**

|  | **Condition of Attraction** | | **Condition of Emotion** | |
| --- | --- | --- | --- | --- |
|  | **Mean** | **SD** | **Mean** | **SD** |
| **AH** | 85.3 | 9.8 | 87.1 | 10.8 |
| **AS** | 23.1 | 23.8 | 69.9 | 14.0 |
| **UAH** | 54.4 | 21.7 | 92.6 | 7.2 |
| **UAS** | 89.9 | 8.2 | 96.4 | 4.9 |

Note: A, “attractive faces,” including both A1 and A2 faces; UA, “unattractive faces,” including both UA1 and UA2 faces; H, “happy faces,” including both H1 and H2 faces; S, “sad faces,” including both S1 and S2 faces.

**Supplementary Table 3. Artifact-free trials in each condition.**

| **Level of**  **Attractiveness** | **Level of**  **Expression** | **Condition of Attraction** | | | | **Condition of Emotion** | | | |
| --- | --- | --- | --- | --- | --- | --- | --- | --- | --- |
|  |  | **Min** | **Max** | **Mean** | **SD** | **Min** | **Max** | **Mean** | **SD** |
| UA1 | S1 | 19 | 32 | 29.2 | 4.2 | 20 | 32 | 29.4 | 4.0 |
|  | S2 | 23 | 32 | 29.7 | 3.4 | 20 | 32 | 29.1 | 3.8 |
|  | H2 | 21 | 32 | 29.4 | 3.7 | 20 | 32 | 29.3 | 3.7 |
|  | H1 | 23 | 32 | 29.2 | 3.3 | 20 | 32 | 29.4 | 3.8 |
| UA2 | S1 | 20 | 32 | 29.6 | 3.6 | 20 | 32 | 29.3 | 3.7 |
|  | S2 | 22 | 32 | 29.4 | 3.2 | 20 | 32 | 29.3 | 4.2 |
|  | H2 | 21 | 32 | 29.5 | 3.7 | 20 | 32 | 29.7 | 3.8 |
|  | H1 | 23 | 32 | 29.7 | 3.5 | 20 | 32 | 29.2 | 4.1 |
| A2 | S1 | 23 | 32 | 29.4 | 3.2 | 20 | 32 | 29.2 | 4.1 |
|  | S2 | 23 | 32 | 29.3 | 3.5 | 20 | 32 | 29.1 | 4.2 |
|  | H2 | 22 | 32 | 29.8 | 3.2 | 20 | 32 | 29.0 | 4.1 |
|  | H1 | 21 | 32 | 29.4 | 3.7 | 20 | 32 | 29.3 | 3.8 |
| A1 | S1 | 24 | 32 | 29.3 | 3.0 | 20 | 32 | 29.1 | 3.8 |
|  | S2 | 21 | 32 | 29.6 | 3.6 | 20 | 32 | 29.6 | 3.7 |
|  | H2 | 24 | 32 | 29.4 | 3.1 | 20 | 32 | 29.5 | 3.6 |
|  | H1 | 22 | 32 | 29.7 | 3.4 | 20 | 32 | 29.3 | 4.0 |

Note: A1 = attractive, A2 = less attractive, UA2 = less unattractive, UA1 = unattractive; H1 = happy, H2 = less happy, S2 = less sad, S1 = sad.
